# Proliferation Increasing Genetic Engineering in Human Corneal Endothelial Cells: A Literature Review

**DOI:** 10.3389/fmed.2021.688223

**Published:** 2021-06-29

**Authors:** Wout Arras, Hendrik Vercammen, Sorcha Ní Dhubhghaill, Carina Koppen, Bert Van den Bogerd

**Affiliations:** ^1^Antwerp Research Group for Ocular Science (ARGOS), Translational Neurosciences, Faculty of Medicine and Health Sciences, University of Antwerp, Wilrijk, Belgium; ^2^Department of Ophthalmology, Antwerp University Hospital, Edegem, Belgium; ^3^Netherlands Institute for Innovative Ocular Surgery (NIIOS), Rotterdam, Netherlands

**Keywords:** genetic engineering, cell therapy, cell proliferation, corneal endothelial cells, corneal endothelial transplant

## Abstract

The corneal endothelium is the inner layer of the cornea. Despite comprising only a monolayer of cells, dysfunction of this layer renders millions of people visually impaired worldwide. Currently, corneal endothelial transplantation is the only viable means of restoring vision for these patients. However, because the supply of corneal endothelial grafts does not meet the demand, many patients remain on waiting lists, or are not treated at all. Possible alternative treatment strategies include intracameral injection of human corneal endothelial cells (HCEnCs), biomedical engineering of endothelial grafts and increasing the HCEnC density on grafts that would otherwise have been unsuitable for transplantation. Unfortunately, the limited proliferative capacity of HCEnCs proves to be a major bottleneck to make these alternatives beneficial. To tackle this constraint, proliferation enhancing genetic engineering is being investigated. This review presents the diverse array of genes that have been targeted by different genetic engineering strategies to increase the proliferative capacity of HCEnCs and their relevance for clinical and research applications. Together these proliferation-related genes form the basis to obtain a stable and safe supply of HCEnCs that can tackle the corneal endothelial donor shortage.

## Introduction

When light enters the eye, the first tissue it passes through is the cornea. This highly specialized transparent tissue is comprised of 5 anatomical layers; the epithelium, Bowman's layer, stroma, Descemet's membrane and finally its most posterior layer, the endothelium. This inner layer of the cornea acts as a leaky barrier that allows the exchange of nutrients and waste products between the corneal stroma and the aqueous humor, but also actively pumps excessive water out of the cornea to maintain a state of relative deturgescence (1). Throughout adulthood, the endothelial cell density (ECD) decreases by 0.3–0.6 % each year because these cells lack the proliferative capacity to compensate for their attrition (2, 3). Human corneal endothelial cells (HCEnCs) are arrested in the G1-phase of the cell cycle due to cell-cell contact inhibition, reduced exposure to growth factors and inhibition of S-phase entry by TGF-β2 in the aqueous humor (4). When these cells are lost or damaged, they rely on a combination of migration and enlargement to preserve the function and integrity of the corneal endothelium (5). Traumatic and congenital pathologies, however, may push these compensatory mechanisms to their limit, causing the cornea to become edematous resulting in a loss of transparency. The current gold standard of treatment is to remove the dysfunctional cell layer and replace it with a corneal endothelial transplant. Due to the corneal donor shortage and lack of banking infrastructures globally, an estimated 12.7 million people are awaiting corneal transplantation worldwide, more than half of which is due to corneal endothelial dysfunction (6).

Over the past decades, different approaches to increase corneal endothelial graft availability have been investigated. Recently, the first results of a clinical trial using a lab-cultured suspension of HCEnCs administered as an intracameral injection was reported in a cohort of patients with endothelial disease with positive results (7). While the effects of this treatment may be altered by the severity of disease at the level of the Descemet membrane (8), it is convincing evidence that such novel cell therapies can be effective (7, 9). Another approach comprises the biomedical engineering of corneal endothelial grafts in the laboratory. As a result, a plethora of corneal endothelial scaffolds have been proposed for use in patients, onto which HCEnCs can be seeded (10, 11). Alternatively, instead of using scaffolds, donor grafts with low ECD counts could also be used for transplantation by increasing the amount of HCEnCs on these grafts (12, 13).

**Figure 1:**
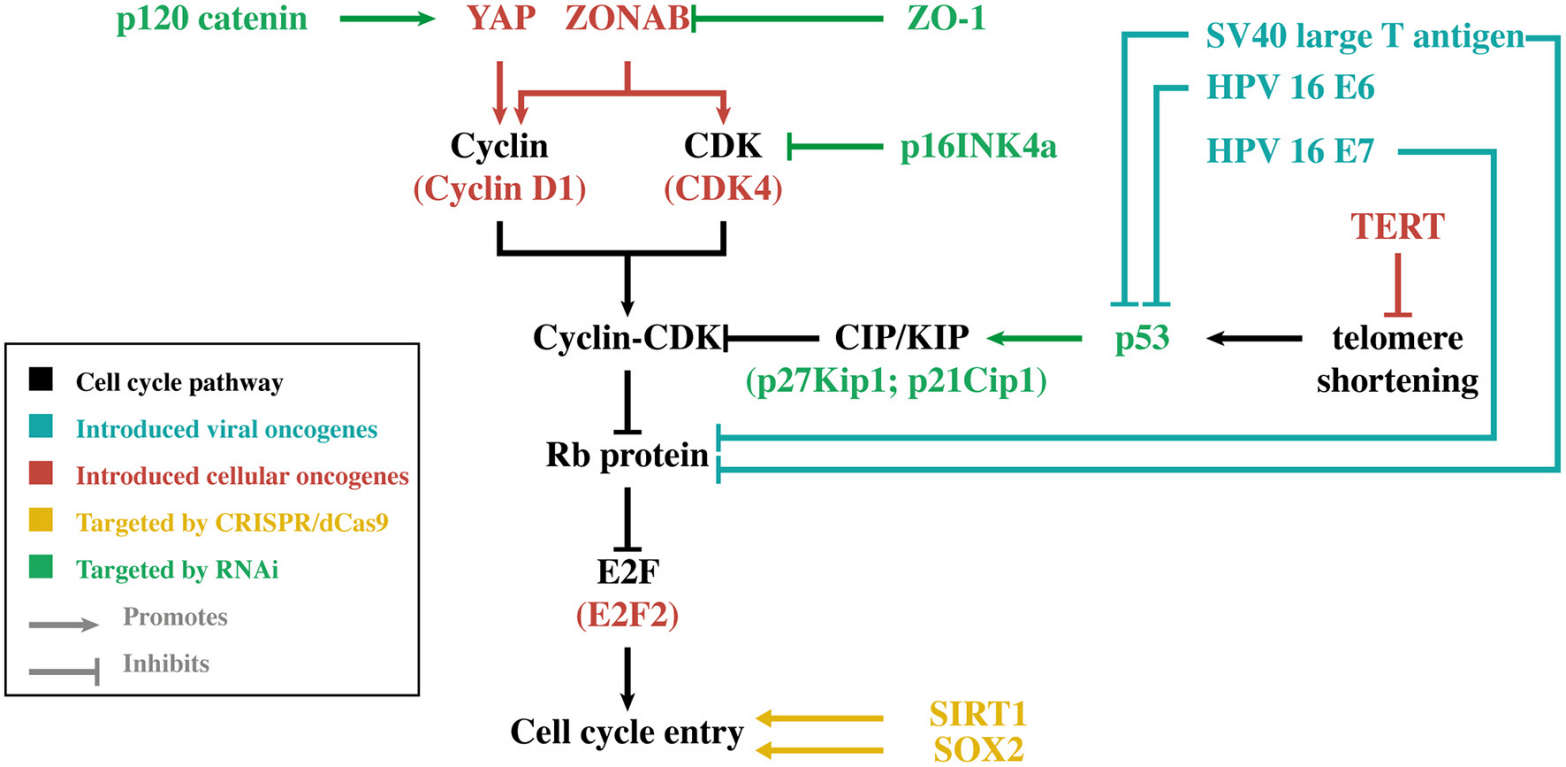
Simplified overview of the main genes discussed in this review. For each gene, its relation to cell cycle entry is illustrated. The different colors indicate the genetic engineering strategy that was used to modify gene expression in human corneal endothelial cells. CDK, cyclin-dependent kinase; CRISPR, clustered regularly interspaced short palindromic repeats; dCas9, deactivated CRISPR-associated protein 9; HPV-16, human papilloma virus type 16; TERT, telomerase reverse transcriptase; Rb, retinoblastoma; RNAi, RNA interference; SOX2, sex-determining region Y-box 2; SV40, simian virus 40; YAP, Yes-associated protein.

Regardless of the approach, the bottleneck of all these strategies remains the limited amount of primary HCEnCs that can only be obtained through standard cell and organ culturing methods. While primary HCEnCs can be cultured *ex vivo*, they can only generate 20–30 population doublings (PD) under standard culturing conditions before becoming senescent (14, 15). The amount of PD that can be obtained is also largely dependent on donor age, as cultures originating from older donors proliferate slower and transform to a senescent phenotype faster (15, 16). In some cell cultures, HCEnCs undergo endothelial-to-mesenchymal transition (EnMT), which can be recognized by the change from their typical hexagonal shape to an elongated morphology, loss of cell-cell contact inhibition and an altered extracellular matrix composition. EnMT also has a detrimental effect on the HCEnC barrier function, rendering them unusable for clinical applications (15). When further optimization of cell culture protocols reaches its limits, genetic engineering may be of benefit. The focus of this alternative approach is to increase the proliferative capacity of HCEnCs without losing their essential characteristics. In general, these genetic engineering strategies are based on viral/cellular oncogene introduction, RNA interference (RNAi) or the clustered regularly interspaced short palindromic repeats (CRISPR)/deactivated CRISPR-associated protein 9 (dCas9) activation system (Figure 1).

## General Overview of Cell Cycle Entry

**Figure 2:**
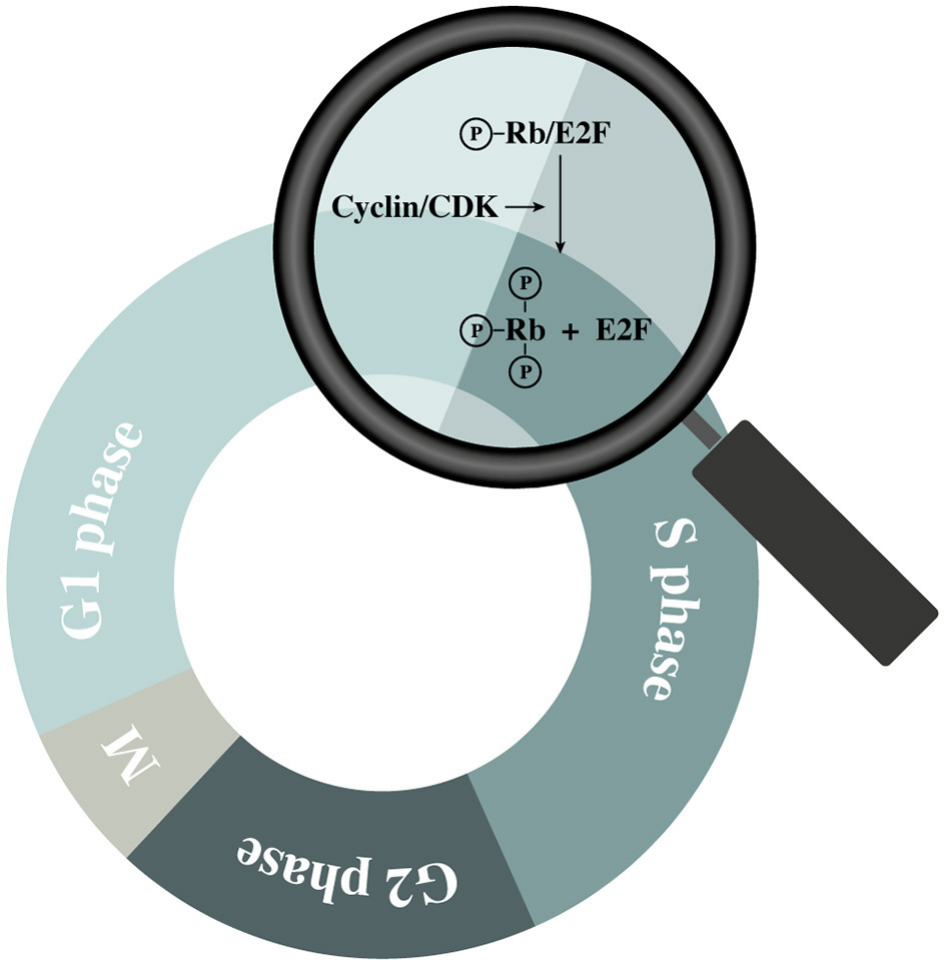
Schematic representation of retinoblastoma (Rb) protein hyperphosphorylation during the G1 phase.

The cell cycle is a tightly regulated process with similar features across all eukaryotic cells. It is regulated by the sequential up- and downregulation of cell cycle related genes where cyclin-dependent kinases (CDKs) play a central role (17). Upon mitogenic activation of cells in G0 or early G1, a chain of events is initiated causing an upregulation of different cell cycle regulating factors including cyclins (18). Cyclins interact with their corresponding CDKs causing the latter enzymes to become activated and phosphorylate their downstream targets. By regulating CDK activities throughout the different phases of the cell cycle, cell cycle-related proteins can be activated in a sequential manner (19). Cyclin D can activate CDK 4 and 6, which induce phosphorylation of the retinoblastoma (Rb) family members (pRb, p107, and p130) (20). In quiescent cells (and in early G1), these proteins are bound to E2F transcription factors, preventing cell cycle progression (18). However, hyperphosphorylation of the Rb proteins diminishes their control over E2F, causing some of the E2F family members to start upregulating their (cell-cycle-associated) target genes (20) (Figure 2). The amounts of active E2F is further increased by a process of positive feedback, which eventually tips the cell over the restriction point. After this “point-of-no-return.” the cell is committed to the following phases of the cell cycle independent of the presence of mitogenic stimuli (18). However, cyclin-dependent kinase inhibitors (CKIs) are also present within the cell. They function as negative regulatory mechanisms that stabilize the G0-phase and induce G1 cell cycle arrest (18, 21). In this group of CKIs, the INK4 proteins and the CIP/KIP protein family can be distinguished based on their structure and specific target. The INK4 family targets specific CDKs (i.e., CDK 4 and 6) while the CIP/KIP family inhibits cyclin-CDK complexes (21). Additionally, p53 is an important suppressor of the cell cycle as it can induce cell cycle arrest, and even apoptosis, in response to the activation of oncogenes or DNA damage. To induce G1-phase arrest, p53 mainly relies on p21CIP1, which prevents the activation of E2F by inhibiting different cyclin-CDK complexes including cyclin D/CDK4 (22).

## Proliferation Enhancement Through Viral Oncogene Introduction

Some viruses have the potential to stimulate the proliferation of mammalian cells by using oncogenes to increase the production of their own genetic material (23). The human viral oncogenes often stimulate proliferation through the inhibition of the tumor suppressor p53 and/or members of the Rb family (24). This capacity to increase the proliferation of a wide range of cells has been employed extensively, but the effect of these oncogenes differs between cell types. While some cells appear unaffected, others exhibit invasive tumorigenic phenotypes with the potential for metastasis (23, 25).

### Simian Virus 40 Large T-antigen

One of the first viral oncogenes used in HCEnCs was a modified simian virus 40 (SV40) early gene region encoding the SV40 large T-antigen (26). This viral protein stimulates cell proliferation by inhibition of p53 and disruption of the Rb-E2F complex through binding with p53 and Rb, respectively. E2F is then free to induce gene transcription needed for cell cycle entry (23). In HCEnCs, SV40 large T-antigen was found to increase the expression of CDK1, CDK2, and CDK4 but also cyclin A and D were upregulated. Conversely, western blotting indicated that the cell cycle inhibitors p27KIP1 and p21CIP1 decreased compared to primary HCEnCs (27).

The expression of SV40 large T-antigen in HCEnCs resulted in an increased proliferation rate and extended survival of HCEnCs from both old and young donors (26–29). Primary HCEnC cultures with low proliferative capacity were found to grow rapidly after expression of the SV40 large T-antigen (29). The cells exhibited a cobblestone-like polygonal morphology at confluence, which differed only slightly from the flatter appearance of unmodified HCEnCs (26, 27). The proliferation rate of transformed HCEnCs decreased when nearing confluence as a result of contact inhibition. However, extended periods of confluence resulted in cell stratification, which is not a feature of the normal corneal endothelium (26). While expression of the SV40 large T-antigen is associated with aneuploidy, chromosomal aberrations in HCEnCs were not routinely assessed (30). However, in these instances where karyotyping was performed, most transformed HCEnCs were found to be diploid (28).

### SV40 Large and Small T-Antigen

SV40 small T-antigen is another SV40-related oncogene, that increases cell proliferation through binding with the protein phosphatase 2A and inhibition of heterochromatin protein 1-binding protein 3 (31). While expression of SV40 small T-antigen alone is not enough to induce transformation, it contributes to SV40 large T-antigen-mediated cell transformation (23, 31).

Expression of both the SV40 large and small T-antigens in HCEnCs resulted in similar outcomes as described for the SV40 large T-antigen alone. However, one culture (obtained from a 91-year-old female donor) recovered from crisis and proliferated indefinitely (>300 PD) while maintaining the characteristic hexagonal shape and size of HCEnCs at confluence (32). Seeding this heterogeneous immortalized HCEnC line (named HCEC-12) onto the denuded Descemet's membrane of a donor cornea *in vitro*, was found to yield a functional endothelial monolayer without exceeding the boundaries of the trabecular meshwork. These cells also exhibited an active pumping capacity, which is a key characteristic of functional HCEnCs (33).

Cells from the HCEC-12 cell line were adapted to serum-free growth conditions and subcloned into two homogeneous immortalized HCEnC lines designated HCEC-B4G12 and HCEC-H9C1. While the phenotype exhibited by the HCEC-B4G12 line resembles that of cultured primary HCEnCs, it was suggested that the HCEC-H9C1 cell line represents transitional HCEnCs due to its atypical phenotype (34). Nowadays, both immortalized cell lines are commonly used in HCEnC research, though not for clinical applications.

### Human Papilloma Virus Type 16 E6/E7

In addition to SV40-related oncogenes, the *E6* and *E7* viral oncogenes of human papilloma virus type 16 (HPV-16) have also been used to increase proliferation of HCEnCs (28, 35). The E6 oncoprotein increases cell proliferation by stimulating the degradation of p53 while E7 induces the ubiquitination of the Rb proteins (36, 37). Aside from promoting the degradation of p53 and the Rb proteins, E6 and E7 also interact with many other cellular factors to increase cell proliferation, which is reviewed elsewhere (38).

Stable expression of both E6 and E7 oncoproteins resulted in immortalization of the HCEnCs while expression of E6 alone only extended their live span by 30 PD (14, 28, 35, 39). Similar to the SV40 large T-antigen expressing HCEnCs, the HPV-16 E6/E7 immortalized cell lines exhibited a cobblestone-like polygonal morphology, were mostly diploid, but also displayed a tendency to form a multilayer when maintained at confluence (28, 35). ZO-1 and N-cadherin mRNA (two popular markers for HCEnC barrier function) were detected, but these results could not be corroborated with immunocytochemical staining of their respective protein and subsequent functional evaluation by means of an Ussing chamber indicated a reduced pump function (35, 39). Subcutaneous injection in nude mice did not result in the formation of solid tumors, indicating that these cells were not tumorigenic (35).

## Proliferation Enhancement Through Cellular Oncogene Introduction

### Human Telomerase Reverse Transcriptase

During cell division, the telomeres at the chromosomal ends of differentiated somatic cells shorten progressively as a result of features inherent to the DNA replication mechanism. Telomeres prevent the cell from recognizing these chromosomal ends as DNA damage. However, excessive cell division shortens the telomeres to such extent that they are no longer able to properly function, causing replicative senescence and apoptosis to occur (40). The replicative senescence that arises is a consequence of p53 activation, which (through p21CIP1) induces cell cycle arrest (41). Telomerase activity is associated with cells that exhibit a high proliferative capacity while it is absent in most differentiated somatic cells (42, 43). Human telomerase reverse transcriptase (TERT), the catalytic subunit of telomerase, can be introduced to prevent the shortening of telomeres. In addition, ectopic *TERT* expression was also found to enhance cellular proliferation in a cell-type dependent manner. Its effect ranges from inducing no increase of cell proliferation, to a limited life span extension or even immortalization (44–46).

In HCEnCs, the introduction of *TERT* did not increase life span for most donors under standard cell culturing conditions (14, 47, 48). However, TERT was found to extend cell survival by approximately 18 PD when culture conditions were adapted to reduce oxidative stress (14). After transfection of the HCEnCs from a 15-day old donor with *TERT*, a subpopulation of fast proliferating cells was found that could be cultured for about 36 PD. These *TERT*-transfected cells exhibited many HCEnC-associated characteristics including contact inhibition, presence of ZO-1 and N-cadherin on protein level and an intact Na^+^/K^+^-ATPase pump function. Furthermore, no aneuploidy or tumorigenicity was observed (47). Schmedt et al. introduced *TERT* into a uniform-appearing subpopulation of cells that was present within a culture of mostly senescent primary HCEnCs. Before *TERT* was introduced, the life span of this subpopulation had already exceeded that of a normal HCEnCs culture. The ectopic expression of TERT caused the cell doubling time to be preserved at higher passages. Extensive characterization of these TERT overexpressing cells did not indicate any adverse effect on HCEnC-associated characteristics and functionality (48).

The relatively low impact of *TERT* on the HCEnC phenotype combined with its telomere lengthening function make it a viable candidate to induce immortalization when combined with another oncogene. The introduction of *TERT* in combination with HPV 16 *E6* or *CDK4* resulted in the immortalization of HCEnCs, while stable expression of CDK4 or HPV 16 E6 alone merely extended their life span. The *TERT*/*CDK4* immortalized HCEnC line remained responsive to contact inhibition and a transcriptome analysis indicated relatively close resemblance to cultured HCEnCs (14).

### CDK4 and Cyclin D1

A variant of *CDK4*, together with the gene coding for cyclin D1 (*CCND1*), has also been introduced in HCEnCs (35). In this CDK4 variant, arginine on position 24 is replaced by cysteine (CDK4R24C) (49). While CDK4R24C has the same functional activity as CDK4, it is not inhibited by p16INK4A and can therefore stimulate cell cycle entry more effectively (50). The cells that stably expressed both CDK4R24C and cyclin D1 were found to have an increased proliferative capacity and cell survival was extended by about 15 PD (35). Their morphology and expression of ZO-1 and N-cadherin mRNA was similar to that of primary cells. Both proteins were also detected by immunofluorescent staining but ZO-1 was particularly prominent around the nucleus instead of being mainly focused at the cellular junctions. Na^+^/K^+^-ATPase driven pump function seemed to be intact, albeit more variable when compared to primary cells, which could indicate a reduced Na^+^/K^+^-ATPase or barrier function. Subcutaneous injection of the transduced cells in nude mice did not result in tumor formation (35).

### E2F2

In contrast to the genetic modifications described above using cells in culture, increasing the ECD by stimulating the proliferation of HCEnCs on their own Descemet's membrane has also been reported (12, 13). Since corneal transplants can be stored in warm organ culture for weeks prior to use (51), *ex vivo* grafts with a low ECD could still be used for transplantation if the ECD is increased. By using adenoviruses, the HCEnCs on full-thickness corneal specimen were transduced with the gene coding for the E2F2 transcription factor (13). As a result, the ECD was found to increase, while the characteristic hexagonal shape and monolayer feature of the HCEnCs were preserved. Since adenoviral-mediated gene introduction only conveys transient gene expression, the number of E2F2 overexpressing cells decreased after 2 weeks (13).

### ZONAB

Another target to increase the proliferative capacity of HCEnCs is the ZONAB/ZO-1 pathway. ZONAB is a Y-box transcription factor that binds to ZO-1 and the cell cycle regulating protein CDK4 (52, 53). As cells are progressing toward confluence, they are known to upregulate ZO-1 expression to form a network of tight junctions (53). However, as ZO-1 is a tight junction associated protein, this causes the amount of cytoplasmic ZONAB to increase at the expense of its nuclear counterpart. Correspondingly, CDK4 was found to be mainly expressed in the cytoplasm and reduced in the nucleus, at confluence (52, 53). ZONAB itself also negatively regulates the *ERBB2* gene, which encodes an oncogenic growth factor receptor (53, 54). However, experiments in a canine kidney cell line indicate that alterations of ERBB2 expression do not influence cell proliferation rates. Therefore, it was suggested that the ZONAB/ZO-1 pathway is more likely to regulate cell cycle arrest through CDK4 (52). ZONAB was also found to have a direct effect on the upregulation of other cell cycle associated genes including the one coding for cyclin D1 (55).

Overexpression of ZONAB in HCEnCs present on the Descemet's membrane of full-thickness corneal specimen increased the ECD significantly, while immunohistochemical staining for F-actin indicated the distinct hexagonal HCEnC morphology. Also the effects of ZO-1 repression, by employing short hairpin RNA (shRNA) targeting ZO-1, have been assessed in HCEnCs but will further be discussed below (12).

## Proliferation Enhancement Through RNA Interference

RNAi-based methods can also be used to enhance cellular proliferation, avoiding the need for introducing oncogenes. With RNAi, specific genes can be downregulated by targeted degradation of their mRNAs while hopefully avoiding unexpected downstream effects.

### ZO-1

Previously, the ZONAB/ZO-1 pathway has been discussed together with the effect of ZONAB overexpression on HCEnC proliferation. In this respect, the downregulation of ZO-1 by employing ZO-1 shRNA has been used with the goal of increasing the proliferative capacity of HCEnCs on a donor graft by exploiting the same pathway. Interestingly, ZO-1 shRNA only increased the amount of HCEnCs significantly on full-thickness donor grafts with a relatively low ECD, independent of age, while this was not reported in the cells overexpressing ZONAB (12). As a result, it was concluded that the ZO-1 downregulated cells were still very sensitive to contact inhibition. Immunohistochemical staining for F-actin and ZO-1 did not indicate disruption of HCEnC barrier but only a reduced expression of ZO-1. The HCEnCs exhibited their characteristic hexagonal to polygonal shape (12). BrdU staining of a ZO-1 siRNA treated contact inhibited monolayer comprised of cultured corneoscleral HCEnCs also indicated an absence of induced proliferation (56). These results indicate that a downregulation of ZO-1 by RNAi is not sufficient to promote HCEnC proliferation in the presence of contact inhibition.

### p53 and CKIs

Other negative regulatory mechanisms of the cell cycle have been targeted to increase the proliferation of HCEnCs aside from the silencing of ZO-1. Targeting of p53 mRNA by stable expression of p53 shRNA was found to increase survival by about 12 PD while combination with TERT overexpression induced immortalization of HCEnCs (14).

Downregulation of the CKI p27KIP1 in a confluent culture by p27KIP1 siRNA caused a 30% increase of ECD in young donors (<28 years) while no increase was observed in HCEnCs originating from older donors (>60 years) (57). The p27KIP1 siRNA transfected cells showed a normal morphology and ZO-1 immunocytochemical staining. Both the use of p27KIP1 siRNA and antisense oligonucleotides was attempted. While both successfully decreased the expression of p27KIP1, p27KIP1 antisense oligonucleotides resulted in a lower survival rate. Therefore, p27KIP1 antisense oligonucleotide-based silencing was not pursued further (57). While p27KIP1 siRNA did not increase the ECD when using cells of older donors, the average amount of HCEnCs between such donors was found to more than double when the expression of p21CIP1 and P16INK4 was simultaneously downregulated by electroporation with their respective siRNA. However, due to extensive variations between the donors, this was not enough to establish a significant difference compared to the control (58).

### p120 Catenin/Kaiso

Cell-cell junctions are important for the maintenance of contact inhibition and barrier function in the HCEnC monolayer (12, 59). The effect of the tight junction associated ZO-1/ZONAB pathway has been discussed, but adherens junctions are also associated with a decreased proliferative capacity of HCEnCs (59). Adherens junctions consist of an extracellular side comprising cadherins that establish cell-cell interactions. On the cytoplasmic side, these cadherins interact with catenins to induce intracellular changes (60). One of these catenins, p120 catenin, both stabilizes E-cadherin and inhibits Kaiso, a transcriptional repressor (61, 62).

Downregulation of p120 catenin (*CTNND1*), by introducing p120 catenin siRNA, decreased the amount of p120 catenin at the cell junction in a contact inhibited monolayer of cultured peripheral HCEnCs (56). Counterintuitively, the amount of nuclear p120 catenin increased through nuclear translocation of this protein, allowing it to inhibit Kaiso (56, 63). This caused the surface area of the HCEnC monolayer to double compared to the control, while maintaining a healthy ECD. This proliferative effect, elicited by p120 catenin/Kaiso signaling, can be partially explained by inhibition of the Hippo pathway (56). The Hippo pathway suppresses cellular proliferation by phosphorylation of transcriptional co-activators Yes-associated protein (YAP) and transcriptional coactivator with PDZ-binding motif (TAZ) (64). The p120 catenin siRNA was found to increase nuclear unphosphorylated YAP and TAZ, allowing them to interact with their (proliferation related) target genes (56). Ectopic expression of YAP in immortalized HCEnCs (B4G12 cell line) induced an overexpression of the previously mentioned cell cycle promoting cyclin D1, which has been identified as a target gene of YAP (65, 66). Cell cycle inhibitors p27KIP1 and p21CIP1 were found to be downregulated (65). It is important to note that the primary HCEnCs from these experiments were not dissociated into single cells by using EDTA-trypsin. Instead, they were isolated while leaving intercellular junctions intact. This was done because disruption of intercellular junctions with EDTA-trypsin, followed by p120 catenin siRNA treatment, negatively influenced proliferation (56).

The apparent relationship between the p120 catenin-mediated Kaiso inhibition and cell proliferation, led to investigating the effect of Kaiso knockdown. Treatment with Kaiso siRNA alone, did not influence nuclear Kaiso expression nor increased BrdU labeling (56). However, Kaiso siRNA was found to work synergistically with p120 catenin siRNA and their combination resulted in a significant expansion of the HCEnC monolayer compared to p120 catenin siRNA alone (56, 63). By combining p120 catenin and Kaiso siRNA, one-quarter of a corneoscleral rim (<1 mm diameter) could be expanded up to 6.8 ± 0.3 mm in diameter, which lies within the range of a transplantable graft. The hexagonal morphology of the cells was preserved and one week after withdrawal from siRNA treatments, immunocytochemical staining indicated similar F-actin, ZO-1 and NA^+^/K^+^-ATPase staining to the control (63).

Weekly p120 catenin and Kaiso siRNA treatment in modified embryonic stem cell medium instead of the supplemental hormonal epithelial medium that was used in the corresponding experiments described above, further increased the proliferative capacity of the HCEnCs. It allowed for the expansion of HCEnCs from one-eighth of the corneoscleral rim to make a graft of 11 ± 0.6 mm in diameter after 5 weeks (67).

## Proliferation Enhancement Through CRISPR/dCas9

The CRISPR/dCas9-system allows the overexpression of endogenous target genes by directing a fusion protein, comprising dCas9 and a transactivation domain, to specific gene promoters through coexpression with guide RNA. The guide RNA determines the target, while the transactivation domain facilitates gene expression (68). This technique has garnered a lot of research attention of late and could be used to increase cell proliferation by enhancing the expression of endogenous oncogenes.

### Sex-Determining Region Y-box 2

Sex-determining region Y-box 2 (SOX2) is a transcription factor that belongs to the SOX family of proteins. The SOX family members are characterized by a specific DNA-binding motif, that allows them to bind to their target genes (69). SOX2 is indispensable for mammalian development, but has also been related to several of the hallmarks of cancer (70). However, it is probably best known as one of the four Yamanaka factors, that were used to convert somatic cells into pluripotent stem cells (i.e., cells able to differentiate into lineages of all three germ layers) (71).

CRISPR/dCas9-mediated overexpression of SOX2 significantly increased cell proliferation and viability in HCEnCs, while maintaining proper ZO-1 expression. Both CDK1 and cyclin D1 were upregulated and expression of the gene coding for p16INK4a was repressed. Interestingly, SOX2 upregulation also caused a repression of COL8A2 (72). Downregulation of the latter has been found to negatively influence HCEnC functionality and proliferation (73). However, this is contradictory to the effects observed with SOX2 overexpression (72). A possible explanation is a difference in the extent of COL8A2 suppression, but it is also likely that other effects of SOX2 upregulation came into play. *In vivo* SOX2 overexpression in a cryoinjured rat corneal endothelium, indicated an increased proliferation and preservation of function by reducing corneal opacification compared to the control. The results suggest that activation of AKT-mediated inhibition of FOXO3a is involved in the increased proliferation elicited by SOX2 in HCEnCs (72). However, aside from its relation to AKT, SOX2 has been found to influence proliferation by interacting with many other proliferation regulation factors (74). Therefore, it is not yet clear which factors are responsible for the observed increase of proliferation in HCEnCs.

### SIRT1

The nicotinamide adenine dinucleotide-dependent deacetylase, SIRT1, is a member of the sirtuin family and has a diverse array of targets inside the cell (75, 76). Its targets comprise both histone and non-histone proteins through which SIRT1 can act as an epigenetic regulator and alter the activity of its target proteins such as p53 (77), c-MYC (78), Rb family members (79), E2F1 (80), FOXO3a transcription factor (81) and more (82). Accordingly, SIRT1 is involved in several cellular processes including proliferation, telomere maintenance, DNA damage response, oxidative stress, apoptosis and energy metabolism (82). However, its effect on proliferation is context dependent since SIRT1 can both promote (83) and limit (84) proliferation of human primary cells. Others also reported no effect of SIRT1 overexpression on replicative lifespan (85).

In HCEnCs, endogenous overexpression of SIRT1 resulted in a significant increase of BrdU staining and cell viability while the polygonal shape of the cells was preserved (86). Correspondingly, cyclin A2 and p16INK4a were upregulated and downregulated, respectively. The same authors also conducted an *in vivo* study on cryoinjured rat corneas in which they found that SIRT1 overexpression decreased corneal opacity and increased ECD (86).

## Discussion

Throughout the years, a variety of genetic engineering strategies have been employed to introduce proliferation related genes into HCEnCs (Table 1). They have increased our understanding of HCEnC proliferation and aid in the search for strategies to expand the amount of these cells for research or clinical purposes. However, comparing proliferation enhancing genes with one another is challenging, because the characteristics that are required will depend on the application. Also the lack of a general consensus about which phenotypical and functional characteristics define a healthy HCEnC monolayer and the diversity of metrics used to quantify proliferation, complicates this matter (87).

**Table 1:** Genes of which the expression has been modified to increase proliferation in HCEnCs. For each gene of which the expression was altered in HCEnCs to enhance cell proliferation, the strategy that was used for genetic engineering and the method of modification is shown.

**Gene(s)**	**Genetic engineering strategy**	**Modification method**	**References**
SV40 early region (SV40 Large T antigen)	Gene introduction	Electroporation	(26)
		Adenovirus	(27, 29)
		Retrovirus	(28)
SV40 early region(SV40 Large and smallT antigen)	Gene introduction	Electroporation	(32)
HPV 16 *E6*	Gene introduction	Retrovirus	(14)
HPV 16 *E6/E7*	Gene introduction	Retrovirus	(28, 35, 39)
*TERT*	Gene introduction	Retrovirus	(14, 48)
		Lipid-based transfection reagent	(47)
*TERT* + HPV 16 *E6*	Gene introduction	Retrovirus	(14)
*p53*	RNAi (siRNA)	Retrovirus	(14)
*TERT* + *p53*	Gene introduction + RNAi (siRNA)	Retrovirus	(14)
*CDK4*	Gene introduction	Retrovirus	(14)
*TERT* + *CDK4*	Gene introduction	Retrovirus	(14)
*CDK4* (CDK4R24C) + *CCND1*	Gene introduction	Retrovirus	(35)
*E2F2*	Gene introduction	Adenovirus	(13)
*ZONAB*	Gene introduction	Lentivirus	(12)
*ZO-1*	RNAi (shRNA)	Lentivirus	(12)
	RNAi (siRNA)	Lipid-based transfection reagent	(56)
*P27KIP1*	RNAi (siRNA)	Lipid-based transfection reagent	(57)
*P21CIP1* + *p16INK4a*	RNAi (siRNA)	Electroporation	(58)
*CTNND1* (p120 catenin)	RNAi (siRNA)	Lipid-based transfection reagent	(56, 63)
*Kaiso*	RNAi (siRNA)	Lipid-based transfection reagent	(56)
*CTNND1* (p120 catenin) + *Kaiso*	RNAi (siRNA)	Lipid-based transfection reagent	(56, 63)
*YAP*	Gene introduction	Lipid-based transfection reagent	(65)
*SOX2*	Crispr/dCas9-mediated upregulation	Lipid-based transfection reagent	(72)
*SIRT1*	Crispr/dCas9-mediated upregulation	Lipid-based transfection reagent	(86)
APst I fragment of simian adenovirus type 7	Gene introduction	Microinjection	(90)
Adenovirus type 5 *E1a/E1b*	Gene introduction	Microinjection	(90)
Adenovirus *E1a* + *HRAS*	Gene introduction	Microinjection	(90)

*CDK, cyclin-dependent kinase; CRISPR, clustered regularly interspaced short palindromic repeats; dCas9, deactivated CRISPR-associated protein 9; HCEnCs, human corneal endothelial cells; HPV-16, human papilloma virus type 16; TERT, telomerase reverse transcriptase; RNAi, RNA interference; SOX2, sex-determining region Y-box 2; SV40, simian virus 40; YAP, Yes-associated protein*.

The ideal proliferation enhancing gene is difficult to define. To make donor grafts with a low ECD available for transplantation, a relatively small increase in proliferation may suffice. However, if the goal is to use these cells for intracameral injection or in combination with endothelial scaffolds, the ideal endpoint would be an off-the-shelf product. While this is not possible for many other transplantable tissues, the immune-privileged environment of the anterior chamber does not require systematic patient/donor matching for corneal endothelial transplantation. Although the current gene engineering strategies allow to only temporarily increase cell proliferation through transient ectopic gene expression, there remain to be concerns with regard to the safety of these methods. Unfortunately, studies concerning the safety of proliferative enhanced HCEnCs are sparse, making it difficult to predict the behavior of these cells *in vivo*.

The extensive global corneal donor shortage demands researchers to find innovative ways to circumvent the 1:1 relation between donor and patient, and thus increase the amount of available donor grafts. Even in countries were no donor shortage existed previously, the COVID-19 pandemic made painfully clear that the supply of corneal grafts could quickly become compromised because of exclusion criteria to prevent transplantation-mediated viral transmission (88, 89). Genetic engineering can offer a solution for this shortage. Many viable proliferation enhancing genes have been proposed, but much more work will be required to assess their value and safety for clinical application.

## Author Contributions

WA searched the literature and wrote the manuscript. WA and BV conceptualized the manuscript and designed figures. BV provided scientific guidance. BV, HV, SN, and CK critically revised and commented the manuscript. All authors have read and approved the final manuscript.

## Conflict of Interest

The authors declare that the research was conducted in the absence of any commercial or financial relationships that could be construed as a potential conflict of interest.

## Abbreviations

CDKs: cyclin-dependent kinases
CKIs: cyclin-dependent kinase inhibitors
CRISPR: clustered regularly interspaced short palindromic repeats
dCas9: deactivated CRISPR-associated protein 9
ECD: endothelial cell density
EnMT: endothelial-to-mesenchymal transition
HCEnCs: human corneal endothelial cells
HPV-16: human papilloma virus type 16
TERT: human telomerase reverse transcriptase
PD: population doublings
Rb: retinoblastoma
RNAi: RNA interference
shRNA: short hairpin RNA
SOX2: sex-determining region Y-box 2
SV40: simian virus 40
TAZ: transcriptional co-activator with PDZ-binding domain
YAP: Yes-associated protein.
